# Multiple etiologies of infectious diarrhea and concurrent infections in a pediatric outpatient-based screening study in Odisha, India

**DOI:** 10.1186/s13099-017-0166-0

**Published:** 2017-04-11

**Authors:** Arpit Kumar Shrivastava, Subrat Kumar, Nirmal Kumar Mohakud, Mrutyunjay Suar, Priyadarshi Soumyaranjan Sahu

**Affiliations:** 1 Infection Biology Laboratory, School of Biotechnology, KIIT University, Bhubaneswar, Odisha 751024 India; 2 Kalinga Institute of Medical Sciences, KIIT University, Bhubaneswar, Odisha 751024 India; 3 Divisions of Pathology, School of Medicine, International Medical University, 57000 Kuala Lumpur, Malaysia

**Keywords:** Diarrhea, Infectious diarrhea, Children, Concurrent infection, Co-infection, Odisha

## Abstract

**Background:**

There are multiple etiologies responsible for infectious gastroenteritis causing acute diarrhea which are often under diagnosed. Also acute diarrhea is one of the major causes of morbidity and mortality among children less than 5 years of age.

**Methods:**

In our study, fecal samples (*n* = *130*) were collected from children (<5 years) presenting with symptoms of acute diarrhea. Samples were screened for viral, bacterial, and parasitic etiologies. Rotavirus and Adenovirus were screened by immunochromatographic tests. Diarrheagenic *Escherichia coli* (EPEC, EHEC, STEC, EAEC, O157, O111), *Shigella* spp., *Salmonella* spp., *Vibrio cholera*, *Cryptosporidium* spp., and *Giardia* spp. were detected by gene-specific polymerase chain reaction.

**Results:**

*Escherichia coli* was detected to be the major etiological agent (30.07%) followed by Rotavirus (26.15%), *Shigella* (23.84%), Adenovirus (4.61%), *Cryptosporidium* (3.07%), and *Giardia* (0.77%). Concurrent infections with two or more pathogens were observed in 44 of 130 (33.84%) cases with a predominant incidence particularly in <2-year-old children (65.90%) compared to children of 2–5 years age group (34.09%). An overall result showed significantly higher detection rates among children with diarrhea in both combinations of two as well as three infections concurrently (*p* = 0.004915 and 0.03917, respectively).

**Conclusion:**

Suspecting possible multiple infectious etiologies and diagnosis of the right causative agent(s) can aid in a better pharmacological management of acute childhood diarrhea. It is hypothesized that in cases with concurrent infections the etiological agents might be complementing each other’s strategies of pathogenesis resulting in severe diarrhea that could be studied better in experimental infections.

**Electronic supplementary material:**

The online version of this article (doi:10.1186/s13099-017-0166-0) contains supplementary material, which is available to authorized users.

## Background

Global and national estimates clearly indicate that diarrheal disease is a major public health concern [1, 2]. According to the World Health Organization (WHO), diarrheal diseases are the second leading cause of death (~760,000 per year) in children <5 years of age. Recent studies suggested that diarrheal diseases are the leading cause of childhood deaths in developing countries. Infectious agents, viz., viruses (Rotavirus, Adenovirus, Hepatitis A&E, Norwalk), bacteria (*E. coli*, *Salmonella*, *Shigella*, *Vibrio*), protozoa (*Giardia*, *Cryptosporidium*, *Cyclospora*, *Microsporidia*, *Isospora*), etc., are usually responsible for serious diarrheal disease outbreaks [3–5]. Among these pathogens, diarrheagenic *E. coli* (DEC), Rotavirus, and * Shigella* spp. are the major contributors of childhood morbidity and mortality [6–8].

Globally Rotavirus is responsible for around 0.5 million-child deaths annually [9], while national estimates from India showed that Rotavirus is responsible for almost 0.1 million deaths, 0.4–0.8 million hospitalization, and 2 million outpatient visits in children <5 years of age [10]. Among DECs, enteropathogenic *E. coli* (EPEC) strain accounts for 5–10% of pediatric diarrhea in resource-poor countries [11]. Shigellosis, caused by enteroinvasive *E. coli* or *Shigella* spp., plays an important role in morbidity and mortality among children <5 years of age. About 125 million cases of endemic shigellosis occur every year in Asian countries [12, 13].

Also past studies from developing countries suggest *Cryptosporidium* to be the leading cause of parasitic diarrhea among children [3, 14], while *Giardia* infects 200 million people each year in developing countries such as Africa, Asia and Latin America [15]. Various studies have elucidated the prevalence of different etiological agents in children with diarrhea [16–18].

Poor hygiene and sanitation condition increases the transmission dynamics of diarrheal diseases in community settings. All these diarrheal agents are transmitted either through contaminated food, water, or through the fecal oral route. However, there are limited numbers of studies focusing on multiple etiologies particularly in children with diarrhea [19, 20].

Concurrent infection with more than single pathogen might be common among children with diarrhea. Also multiple infections of diarrheal pathogens might cause more severe diarrhea compared to infection with a single pathogen, thereby complicating the treatment management procedures [17, 21]. Due to scarcity of reports on concurrent infections causing diarrhea among children from the studied province in India, we attempted to estimate the overall burden of major diarrheal pathogens and possible concurrent infections with multiple infectious agents among children in a hospital-based screening study.

## Methods

### Study design

This study was conducted in a tertiary care teaching hospital in Bhubaneswar, Odisha (India). Fecal samples were collected from 130 children from June 2015 to April 2016. Stool samples were collected from children considering the inclusion and exclusion criteria. Inclusion criteria were children less than 5 years of age and 3 or more than 3 diarrheal episodes in a day. Children having malabsorption, immunocompromised individuals, and patients who have undergone immune suppressive therapy and prolonged steroid treatment were excluded from our study. Fecal samples were also collected from 48 non-diarrheal children less than 5 years of age, which acted as a control group. The study protocol was reviewed and approved by the Institutional Ethics Committee. Informed consent and patient datasheets were maintained for each participant.

### Sample collection and DNA extraction

From each of these participants, fecal samples were collected in a sterile vial. Samples were placed on ice and transported to lab within 4 h and processed immediately. Total fecal genomic DNA was extracted from stool using QIAamp Fast DNA stool Mini Kit (Qiagen, Germany) according to the manufacturer’s instruction. The quantity and purity of extracted DNA were measured using a Nanodrop (Epoch BioTek, USA).

Extracted DNA samples were stored at −20 °C until further processing. Bacterial genomic DNA was isolated from pure cultures of *S. enterica* subsp*., serovar typhimurium*, *V. cholera*, *S. flexneri*, and *E. coli* (from the repository) using blood and tissue genomic DNA isolation kit (Qiagen, Germany) as per the manufacturer’s protocol. These were used as positive controls for pathogen detection by polymerase chain reaction (PCR).

### Immunochromatographic test for detecting viral pathogen

Fecal specimens were screened for the presence of Rotavirus and Adenovirus by using immunochromatographic test (*Combi*-*Strip C*-*1004, Coris Bioconcept ltd*- *Belgium*) in accordance with the manufacturer’s instructions. This is a rapid diagnostic test based on homogenous membrane system with colloidal gold particles. The test was carried out as described previously [22].

### PCR assay for detecting bacterial and parasitic pathogens

Fecal genomic DNA extracted from stool was used as a template in a series of PCR amplification reactions using primers specific for each pathogen (Table 1). Bacteria-specific genomic DNA isolated from pure cultures was used as a positive amplification control in PCR screening. For detecting protozoan parasites, positive DNA control for *Cryptosporidium* was obtained from Christian Medical College, Vellore (India), while standard *Giardia* DNA was obtained from the Institute of Parasitology, University of Zurich. PCR cycling conditions for different bacterial and protozoan parasites were as follows: Initial denaturation at 95 °C for 5 min, followed by 34 cycles of denaturation of 94 °C for 30 s, annealing at primer-specific temperature at 30–45 s and extension at 72 °C for 1 min, and final extension for 72 °C for 7 min. All PCR products were subjected to 1–1.5% agarose gel electrophoresis to confirm positive samples.

**Table 1:** Detailed description of pathogen-specific polymerase chain reaction primers for their specific detection in stool

Pathogen	Target gene	Primer name	Primer sequence (5′ to 3′)	Tm (°C)	Size (bp)	References
*Escherichia coli*						
EAEC	EAEC pCVD432	EAEC_pCVD432_F_11	CTGGCGAAAGACTGTATCAT	51	630	[23]
		EAEC_pCVD432_R_12	AAATGTATAGAAATCCGCTGTT			
EPEC	EPEC bfpA	EPEC_bfpA_F_9	TTCTTGGTGCTTGCGTGTCTTTT	53	367	[23]
		EPEC_bfpA_F_10	TTTTGTTTGTTGTATCTTTGTAA			
EPEC	EPEC eae gene	EPEC_eaeA_F	GACCCGGCACAAGCATAAGC	59	384	[24]
		EPEC_eaeA_R	CCACCTGCAGCAACAAGAGG			
STEC	EHEC vt1	EHEC_vt2_R_7	ACCGTTTTTCAGATTTT(G/A)CACATA	52	298	[23]
		EHEC_vt2_R_8	TACACAGGAGCAGTTTCAGACAGT			
STEC	EHEC hlyA gene	EHEC_hlyA_F	GCATCATCAAGCGTACGTTCC	57	534	[24]
		EHEC_hlyA_R	AATGAGCCAAGCTGGTTAAGCT	54	180	
STEC	stx1	*E. coli*_stx1F	ATAAATCGCCATTCGTTGACTAC			[24]
		*E. coli*_stx1R	GGCACTGTCTGAAACTGCTCC			
STEC	stx2	*E. coli*_stx2F	GGCACTGTCTGAAACTGCTCC	56	255	[24]
		*E. coli*_stx2R	TCGCCAGTTATCTGACATTCTG			
STEC	rfbE 157:H7	O157_F	CGGACATCCATGTGATATGG	52	259	[24]
		O157_R	TTGCCTATGTACAGCTAATCC			
STEC	H7 FLIC	H7_FLIC-R_13	GCGCTGTCGAGTTCTATCGAGC	59	625	[23]
		H7_FLIC-R_14	CAACGGTGACTTATCGCCATTCC			
STEC	O111 rfb region	O111_F	TAGAGAAATTATCAAGTTAGTTCC	49	406	[24]
		O111_R	ATAGTTATGAACATCTTGTTTAGC			
*Shigella* spp.						
	ShET 1	*Shigella*_1A_F	CAG CGT CTT TCA GCG ACA GTG TTT	57	530	[8]
		*Shigella*_1A_R	AGC ATG ATA CTC AAC AGC CAG ACC			
		*Shigella*_1B_F	ATA CTG GCT CCT GTC ATT CAC GGT			
		*Shigella*_1B_R	GGA AGT GAC AGG GCA TTT GTG GAT			
*Salmonella* spp.						
	Sdf I	*Salmonella*_sdf1_FW	TGT GTT TTA TCT GAT GCA AGA GG	53	304	[25, 26]
		*Salmonella*_sdf1_RW	TGA ACT ACG TTC GTT CTT CTG G			
*Vibrio cholera*						
	Vc	*Vibrio*_Vc_FW	GTTCGCGCTGGTGAAGGTTCA	57	192	[27]
		*Vibrio*_Vc_RW	TGGCATACCAGAGTCTTTCTGTG			
*Cryptosporidium* spp.						
	18 s SSU rRNA locus	18 s Morgan F	AGTGACAAGAAATAACAATACAGG	60	298	[28]
		18 s Morgan R	CCTGCTTTAAGCACTCTAATTTTC			
*Giardia* spp.						
	gdh gene	GDHeF	TCAAGCTYAAYGCYGGYTTGCGT	57	432	[29]
		GDHiF	CAGTACAACTCYGCTCTCGG			
		GDHiR	GTTRTCCTTGCACATCTCC			

### Statistical analysis

To examine associations between the presence or absence of diarrheal pathogen and risk factors within the different age groups (<2 or ≥2 years) and different multiple infections in children, logistic regression was used. Residence location (rural or urban) was considered for their possible association with the occurrence of diarrheal pathogen infections. Descriptive statistics was done to calculate odds ratios and 95% confidence intervals (CI), and p values were used to infer statistical associations based on one-tailed *t* test (Mantel–Haenszel Chi-square statistics) using free statistical software Epi-Info (http://www.openepi.com).

## Results

In the present study, overall result showed the highest detection rate for diarrheagenic *E. coli* (DEC) followed by Rotavirus and *Shigella* spp. in cases with symptoms of diarrhea (Table 2). Besides DEC (30.7%), the other pathogens with a decreasing order of detection rates were Rotavirus (26.15%), *Shigella (*23.84%), Adenovirus (4.61%), *Cryptosporidium* (3.07%), and *Giardia* (0.7%). Different strains of DEC such as EPEC (21.53%), STEC (10.76%), EAEC (6.90%), 0157 (4.61%), and EHEC (0.77%) were detected in the stool samples from cases. All these samples were negative for *Salmonella* spp. and *Vibrio cholera.* Surprisingly DEC, *Shigella* spp. and Adenovirus were also detected in 20.83, 4.61, and 2.08% of the healthy control subjects, respectively (Table 2). Representative gel image and immunochromatographic test positive strips are shown in Additional file 1.

**Table 2:** Frequencies of detection of etiological agents causing diarrhea in children

Infectious agent detected	Method of detection	Frequency (%) of detection in		*p* value
		Diarrheal group (*n* = 130)	Non-diarrheal group (*n* = 48)	
DEC^a^	PCR	40 (30.7)	10 (20.83)	0.1935
STEC	PCR	14 (10.76)	3 (6.25)	0.3685
EPEC	PCR	28 (21.53)	5 (10.41)	0.0975
EHEC	PCR	1 (0.77)	0	0.9434
EAEC	PCR	9 (6.9)	1 (2.08)	0.2412
O 157	PCR	6 (4.61)	1 (2.08)	0.4525
*Shigella* spp.	PCR	31 (23.84)	2 (4.16)	0.0086*
Rotavirus^b^	Immunochromatography	34 (26.15)	0	0.0135*
Adenovirus^b^	Immunochromatography	6 (4.61)	1 (2.08)	0.4525
*Cryptosporidium* spp.	PCR	4 (3.07)	0	0.4091
*Giardia* spp.	PCR	1 (0.77)	0	0.3652

* Statistically significant

^a^ DEC includes all detected strains of diarrheagenic *E. coli*. Each of those strains were diagnosed using strain-specific primers as recommended in the previously published reports (see details as shown in Table 1)

^b^ Rotavirus and Adenovirus were detected by Immunochromatography test using commercially procured kit (*Combi*-*Strip C*-*1004, Coris Bioconcept ltd*- *Belgium*)

Among the control subjects for which DEC was detected, the majority had infections with EPEC (10.41%) followed by STEC (6.25%), EAEC (2.08%), and O 157 (2.08%). No healthy subject was detected with Rotavirus, *Giardia, Cryptosporidium* spp.*, Salmonella* spp., and *Vibrio* spp. The pathogen detection rates for cases and controls for each pathogen category are statistically analyzed which showed a significantly higher detection rate for *Shigella* spp (*p* = 0.0086) and Rotavirus (*p* = 0.0135), whereas the differences were not significant for rest of the detected pathogens.

Gender-wise distributions of diarrheal incidences for different etiological agents are shown in Table 3. The total detection rate for at least one infectious etiology among the cases with acute diarrhea was slightly lower in male patients (54.28%) than in female patients (60%). Only Rotavirus detection was significantly higher among male cases (*p* = 0.0003). Among all the cases with diarrhea, detection rate of DEC was slightly higher among males (31.42%) compared to females (30%). *Cryptosporidium*, *Shigella*, and *Giardia* were also detected with relatively higher rate in males compared to females. However, Adenovirus detection rate was moderately higher among females though the difference was not significant on statistical analysis.

**Table 3:** Gender-wise distribution of incidences of infectious etiologies of diarrhea in children

Total no of samples	Total no. with an infectious diagnosis	No (%) of cases tested positive for different etiologies					
		*Cryptosporidium*	Adenovirus	Rotavirus*	*Shigella* stx	*Giardia*	DEC
Male							
70	38 (54.28)	3 (4.28)	2 (2.85)	28 (40)	20 (28.57)	1 (1.42)	22 (31.42)
Female							
60	36 (60)	1 (1.66)	4 (4.66)	6 (10)	11 (18.33)	0 (0)	18 (30)
Statistics							
*p* value	0.5121	0.406	0.3158	0.0003	0.1749	0.5589	0.8604
95% CI (lower, upper)	0.3937, 1.5919	0.2675, 26.0903	0.0727, 2.3316	2.2755, 15.8209	0.7734, 4.1051	0.1044, 65.3068	0.5062, 2.2595
Odds ratio	0.7917	2.642	0.4118	6	1.7818	2.6115	1.0694

Percentages are calculated based on the total number of male and female cases with symptoms of diarrhea as included in the present study (*n* = 70 and 60, respectively)

*DEC* diarrheagenic *Escherichia coli*

* Statistically significant

Among cases with diarrhea, at least one infectious etiology was detected in 24 of 40 (60%) children living in rural settings in comparison to 50 of 90 (55.55%) children from urban settings. Adenovirus was more prevalent in rural children (*p* = 0.026) (Table 4). *Cryptosporidium* was also detected with higher rate among rural children when compared to urban ones (*p* = 0.02623). Though DEC was also detected predominantly in rural children, the difference however was not significant (*p* > 0.05). Among DEC, only STEC was found significantly higher (*p* = 0.014) in rural children (Additional file 2). In contrast, Rotavirus was more prevalent in urban children (*p* = 0.027). *Shigella* and *Giardia* detection rates were only slightly higher among the urban in comparison to rural children but not statistically significant (*p* > 0.05).

**Table 4:** Location-wise distribution of incidences of infectious etiologies of diarrhea

Total no of samples	Total no. with an infectious diagnosis	No (%) of cases tested positive for different etiologies					
		Cryptosporidium*	Adenovirus*	Rotavirus*	*Shigella* stx	*Giardia*	DEC
Rural							
40	24 (60)	3 (7.5)	4 (10)	6 (15)	9 (22.5)	0 (0)	16 (40)
Urban							
90	50 (55.55)	1 (1.11)	2 (2.22)	28 (31.11)	22 (24.45)	1 (1.11)	24 (26.66)
Statistics							
P value	0.3190	0.02623*	0.0260*	0.0273*	0.4055	0.2525	0.0650
95% CI (lower, upper)	48.33, 65.12	0.9403, 7.907	1.921, 9.915	19.33, 34.34	17.3, 31.89	0.0, 4.657	23.46, 39.18
Odds ratio	1.2	7.216	4.889	0.3908	0.8974	0	1.833

Percentages are calculated based on the total number of rural and urban children with symptoms of diarrhea as included in the present study (*n* = 40 and 90, respectively)

*DEC* diarrheagenic *Escherichia coli*

* Statistically significant

The data on age group (<2 years, and 2–5 years)-wise distribution of various etiological agents in children presenting with symptoms of acute diarrhea are depicted in Table 5. We observed that Rotavirus*, Shigella*, and DEC were the three major etiological agents detected in children under both the age groups. Cases positive for at least one infectious etiology were higher among children <2 years of age (58.53%) in comparison to those between 2 and 5 years (54.16%) which was not significant though (*p* > 0.05). Of all etiologies, only Rotavirus infection was found significantly associated with children under <2 years age group (*p* = 0.003).

**Table 5:** Age group-wise distribution of incidences of different etiological agents causing diarrhea in children in diarrheal group

	No (%) of cases under age groups		Statistical analysis	
	<2 years (*n* = 82)	2–5 years (*n* = 48)	*p* value	Odds ratio (95% CI)
At least one infectious etiology	48 (58.53)	26 (54.16)	0.316	1.195 (0.582, 2.449)
*DEC*	22 (26.82)	18 (37.49)	0.106	0.611 (0.285, 1.309)
*Shigella flexneri*	20 (24.39)	11 (22.91)	0.429	1.085 (0.468, 2.515)
*Rotavirus*	28 (34.14)	6 (12.49)	0.003**	3.63 (1.377, 9.57)
*Adenovirus*	2 (2.43)	4 (8.33)	0.079	0.275 (0.048, 1.562)
*Cryptosporidium*	4 (4.87)	0	0.076	Undefined#
*Giardia*	1 (1.21)	0	0.315	Undefined#

Percentages are calculated based on the total number of children with symptoms of diarrhea under <2 years and 2–5 years age groups (*n* = 82 and 48, respectively)

** Extremely statistically significant

Among the different DEC strains, EPEC was detected significantly higher in <2 years children in comparison to >2 years age group (p = 0.001) (Table 6), while no significant differences were observed on detection rates of STEC and EAEC when compared between the above two age groups. Moreover, *E. coli* O157 was detected with higher frequency among <2-year-old children, although statistically not significant (*p* < 0.28). *Cryptosporidium*, *Giardia*, and *E. coli* O157 were observed more predominantly in children >1 year of age (*data not shown*).

**Table 6:** Age group-wise distribution of incidences of different strains of Diarrheagenic *E. coli* causing diarrhea in children

	No (%) of cases under age groups		Statistical analysis	
	<2 years (*n* = 22)	2–5 years (*n* = 18)	*p* value	Odds ratio (95% CI)
*STEC*	7 (31.81)	7 (38.88)	0.328	0.733 (0.198–2.704)
*EPEC*	20 (90.9)	8 (44.44)	0.001**	12.5 (2.226–70.18)
*EHEC*	1 (4.54)	0	0.275	Undefined#
*O 157*	4 (18.18)	2 (11.11)	0.288	1.778 (0.286–1.04)
*EAEC*	5 (22.72)	4 (22.22)	0.635	1.56 (0.243–10.03)

Percentages are calculated based on the total number of children with symptoms of diarrhea with detection of DEC under <2 years and 2–5 years age groups (*n* = 22 and 18, respectively)

** Extremely statistically significant

**Fig. 1:**
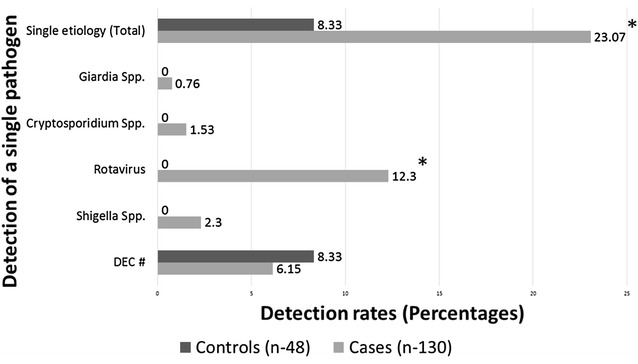
Bar graphs showing detection rates of infections with a single etiological agent among children presenting with diarrhea in comparison to non-diarrheal controls. In a 2 × 2 table analysis for comparison of proportions between cases and controls for cases with detection of a single infection, all expected values (row total X column total/grand total) were ≥5. So Chi-square analysis was recommended. Two-tailed *p* value by Mantel–Haenszel Chi-square test = 0.02680. For single infection with Rotavirus, at least one expected value (row total X column total/grand total) was <5. So Mid-P exact test was recommended rather than Chi-square (http://www.openepi.com). Two-tailed *p* value by Mid-P exact test = 0.005014. *Statistically significant.# DEC: any strain of diarrheagenic *E. coli*

The detection rates and their distribution among each individual pathogen type (only a single pathogen detection) comparing cases and controls are shown in the bar graphs as in Fig. 1. There was a significantly higher rate of detection only for Rotavirus mono-infection among cases with diarrhea in comparison to the controls (*p* = 005014). Also the overall rate of detection of a single infection only was significantly higher among cases in comparison to control subjects (*p* = 0.0268).

In this study, we observed many children infected with multiple pathogens, and the detection rates under different combinations of concurrent infections are presented in Table 7. Overall result showed simultaneous detection of two or more pathogens in 30% of cases included in this study. Of a total 44 cases with co-infections, 33 had double infections, 10 had triple infection, and only 1 had infection with more than three pathogens concurrently. Surprisingly, in case of control group of 48 children with no diarrhea, co-infections were detected in 3 (6.25%) children.

**Table 7:** Co-infection combinations with two or more infectious etiologies detected in diarrheagenic group vs controls

Combinations of pathogens	Frequency (%) of detection cases (*n* = 130)	Frequency (%) of detection controls (*n* = 48)	Statistical analysis
Double infections			
*Shigella*+STEC	5 (3.84)	0	Two-tailed *p* value by Mantel–Haenszel Chi-square test = 0.004915*
Shigella+EPEC	3 (2.3)	0	
*Cryptosporidium*+EPEC	1 (0.76)	1 (2.08)	
EPEC+O157	1 (0.76)	1 (2.08)	
Rotavirus+*Shigella*	7 (5.38)	0	
Adenovirus+EPEC	2 (1.53)	0	
Adenovirus+*Cryptosporidium*	1 (0.76)	0	
Rotavirus+EPEC	6 (4.61)	0	
*Shigella*+O157	2 (1.53)	0	
*Shigella* +EAEC	2 (1.53)	0	
Adenovirus+EAEC	1 (0.76)	0	
Rotavirus+EAEC	1 (0.76)	0	
EPEC+EAEC	2 (1.53)	0	
EPEC+STEC	0	1 (2.08)	
Double infection (total)	33 (25.38)	3 (6.25)	
Triple infections			
*Shigella*+STEC+EPEC	2 (1.53)	0	Two-tailed *p* value by Mid-P exact test = 0.03917*
EPEC+EHEC+O157	1 (0.76)	0	
Rotavirus+*Shigella*+STEC	2 (1.53)	0	
Rotavirus+*Cryptosporidium*+EPEC	1 (0.76)	0	
Rotavirus+*Shigella*+EPEC	1 (0.76)	0	
Rotavirus+*Shigella*+EPEC	1 (0.76)	0	
*Shigella*+STEC+EPEC	1 (0.76)	0	
Adenovirus+*Shigella* +EPEC	1 (0.76)	0	
EAEC+O157+EAEC	1 (0.76)	0	
Triple infection (total)	10 (7.69)	0	
Adenovirus+*Shigella*+STEC+EPEC+O157+EAEC	1 (0.76)	0	Two-tailed *p* value by Mid-P exact test = 0.7303
Combination of Pathogens (more than three infections)			

Statistical analysis is shown considering only total number of cases and control subjects and respective co-isolation rates (double, triple, or more than three infections). In a 2 × 2 table analysis for comparison of proportions between cases and controls for double infections group, all expected values (row total X column total/grand total) were ≥5. So Chi-square analysis was recommended. For triple infections group and more than three infections one, at least one expected value (row total X column total/grand total) was <5. So Mid-P exact test was recommended rather than Chi-square (http://www.openepi.com)

*n* total number of subjects

* Statistically significant

An overall analysis showed statistically significant differences between detection rates for combinations of two infections concurrently among cases vs controls (*p* = 0.004915). Co-infection of Rotavirus with *Shigella* was the most frequent combination, which was detected in 5.38% cases, followed by Rotavirus with EPEC (4.61%) and *Shigella* with STEC (3.84%). Co-infections with detection of two pathogens were detected in one healthy control child each with the following combinations, viz., EPEC with *Shigella*, EPEC with STEC, and EPEC with O 157.

Overall analysis also showed statistically significant differences between detection rates for combinations of three infections concurrently among cases vs controls (*p* = 0.03917). There were two cases each under the following combinations with triple infection: (1) Rotavirus and *Shigella* with STEC; (2) *Shigella* and STEC with EPEC. Other combinations of triple infections were detected only in one child in each combination of concurrent infections (Table 7).

**Fig. 2:**
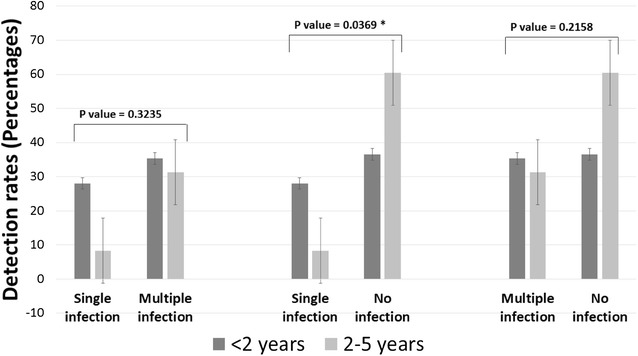
Bar graphs comparing detection rates of mono-infections and co-infections among children of different age groups. Data were analyzed considering the cases with no infectious agent detection as control groups (30 cases under <2 years age group, and 26 cases under 2–5 years age group). In 2 × 2 table analysis for comparison of proportions between cases with detection of single/multiple infections in both age groups, all expected values (row total X column total/grand total) were ≥5. So Chi-square analysis was recommended. Two-tailed *p* value by Mantel–Haenszel Chi-square test = 0.0369 for single infections. Two-tailed *p* value was 0.2158 for multiple infections. When the data were compared between singe vs multiple infections under both age groups, the two-tailed *p* value was 0.3235. *Statistically significant

Age group-wise distributions of mono-infections and multiple infections are shown in Fig. 2. Of the total 30 cases with detection of a single infection, there was a predominance under the younger age group (<2 years) children. Similar observation was recorded in case of multiple infections where majority was detected in the younger age group. There was no statistical difference when mono- and multiple infections were analyzed vs two age groups (<2 years and 2–5 years). When data were analyzed considering the cases with no infectious agent detection as control groups (30 cases under <2 years age group, and 26 cases under 2–5 years age group), two-tailed *p* value by Mantel–Haenszel Chi-square test = 0.0369 for single infections showed a significant difference between the two age groups for single infection.

Overall data on disease severity (only in terms of numbers of liquid motions per day) compared between two groups, i.e., cases with detection of single infection vs cases with detection of more than one infections, are presented in Table 8. Two-sample independent *t* test analysis revealed significantly higher number of motions per day among cases with multiple infections compared to those with single infection based on both equal variance (*p* = 0.001015) and unequal variance (*p* = 0.006885). Other major associated symptoms were vomiting (46%) and fever (23%); when the symptoms were correlated with the test positivity for different etiologies, majority of cases positive for *Shigella* and Rotavirus had vomiting (29%) and fever (27%) (*data not shown*).

**Table 8:** Comparison of the role of single infection vs multiple infections on average motions per day

	n	No. of motions/day (min–max)	Mean average no	SD	Two-sample independent t test
Single infection	30	2–30	10.71	12.6	Equal variance (0.001015)**
More than one infections	44	12–25	17.5	3.2	Unequal variance (0.006885)**

*n* total number of cases

*min* minimum, *max* maximum

Two-sample independent t test using OpenEpi (Version 3), open source calculator revealed significantly higher number of motions per day among cases with multiple infections compared to those with single infection based on both equal variance (*p* = 0.001015) and unequal variance (*p* = 0.006885)

** Statistically extremely significant

## Discussion

Infectious diarrhea is a frequent problem in low-income countries which is a leading cause of death among children under 5 years of age [3]. Though many different types of agents are reported to be associated with infectious diarrhea, DEC has however been known to be the most commonly diagnosed etiology in India [30]. The overall result in our study showed that 56.92% of the children with diarrhea were diagnosed to be positive for at least one infectious etiology among the children with diarrhea. A study in the past showed *E. coli* (different strains) to be responsible for as much as 25% of all diarrheal diseases in developing countries [31]. In this study, we also found DEC to be the most common diarrheal agent in the study population.

In the diarrheal group, more than 1 infectious agent was diagnosed in 44 (33.84%) cases. In our study, we found Rotavirus+*Shigella* to be the most frequent co-infection combination, while a previous study from Leon Nicaragua reported EAEC along with EPEC to be the most frequent co-infection [23].

The number of males suffering with diarrheal diseases was slightly more in comparison to females, which is similar to another study from India [30]. Our study showed that at least one infectious etiology in diarrhea is more frequently detected among children living in rural settings than urban settings. This may be due to the poor hygiene and sanitation conditions of rural population that posses a high risk of infection among children as recommended earlier.

In this study, the major etiological agent was identified to be DEC followed by Rotavirus and *Shigella* in the age group of <2 years. Study elsewhere showed DEC being responsible for acute diarrhea in 30–40% of the affected children [31]. EPEC, EAEC, and ETEC were the most common strains of *E. coli* detected in diarrheal diseases as reported elsewhere [23]. In our study, EPEC was found more frequent than other DEC pathotypes, while in other studies EAEC was detected more frequently [32]. Few studies have also highlighted the role of *Shigella* as an important etiological agent for diarrheal disease [33, 34]. In our study, we observed that *Shigella* was responsible for 23.84% of infection in children with acute diarrhea.

Rotavirus infection was associated with substantial hospitalization and deaths among children [7]. In our study, we observed Rotavirus as the second leading cause of childhood diarrhea. Rotavirus was also present as multiple mixed infections with other diarrheal pathogens. Previously in a study from China, co-occurrence of Rotavirus and *Sapovirus*, *Astrovirus*, ETEC, or *Campylobacter jejuni* was observed in children [35]. In this Chinese study, co-infections between pathogens were found to be common; especially the two pairs, Rotavirus and Adenovirus, and *Norovirus* GII and *Salmonella* were reported to be positively associated with diarrhea.

*Cryptosporidium* and *Giardia* were shown to affect children younger than five years of age in India [36]. In this study, we observed that 4.61% of diarrheal cases were due to *Cryptosporidium* and 0.77% of cases ware due to *Giardia*. *Giardia lamblia* was not significantly associated with diarrhea in a study from Bangladesh [37].

Few studies from Africa have reported that almost one-third of studied children were infected with bacterial pathogens and about ten percent of them were co-infections [17, 38]. In our study, we observed that about 65.90% of cases have multiple infection in children <2 years of age. We could not observe any statistical significance among mono-infections and multiple infections with respect to age group between <2 years of age and 2–5 year age group (*p* = 0.32). A recent study from China reported viral–bacterial co-infection in <5-year-old children [35]. In our study, we observed several mixed infections such as DEC with *Shigella*, Rotavirus with EPEC, and Rotavirus with *Shigella*. We observed that especially with Rotavirus and Adenovirus co-infections there was an increase in the diarrheal episodes per day.

Rotavirus co-infection with other enteric bacterial and protozoan pathogens was reported previously [39]. In our study, mixed infections of *Cryptosporidium* with *Rotavirus*, Adenovirus, and EPEC were observed. *Cryptosporidium* infection was more frequently observed in <2 year age group in comparison to 2–5 year age group, although this was not statistically significant. We could not observe any association between *Cryptosporidium* infection along with vomiting and fever in the infected individuals. A synergistic effect between Rotavirus and other co-infecting pathogen(s) on diarrheal disease was also reported from a Latin American study [21].

Most studies of the causes of diarrhea in low-income and middle-income countries have looked at severe disease in people presenting for care, and there are few estimates of pathogen-specific diarrheal burdens in the community. In a recent cohort study, a substantial heterogeneity in pathogen-specific burdens of diarrhea was identified where the most important determinants were revealed to be age, geography, season, vaccine usage, and symptoms [40]. Community screening was beyond scope of the present study. However, considering disease severity to be the most important criteria to access the possibility of a synergistic effect due to mixed infection in childhood diarrhea, in our study we could note an increased frequency of diarrheal episodes in cases that were diagnosed to have multiple infectious etiologies. A recent study from China also supports the above fact where virus–bacteria and virus–parasite co-infections are the aggravating factors of severe diarrhea in children [39].

There are limited number of studies in India, which can really enlighten on acute childhood diarrhea and far lesser in number when it comes to its bacterial enteropathogenesis. However, *E. coli* has been recorded as the predominant bacteria followed by *Shigella*, *Salmonella*, *Klebsiella*, and *Campylobacter* [29]. A study from Bangladesh identified *C. jejuni*, ETEC, *Shigella* spp., and *V. cholerae* O1 as major bacterial pathogens that are significantly associated with diarrhea [37], while in our study EPEC and *Shigella* were found to be the most common diarrheal etiological agents.

Earlier studies support increased severity of diarrhea in the presence of Rotavirus-*E. coli* co-infection [41, 42]. Simultaneous interaction of Rotavirus and *E. coli* or Rotavirus and *Shigella* results in a greater risk of having diarrhea than would be expected if the co-infected organism acted independently of one another [21]. Similar co-infection patterns of Rotavirus with *E. coli* and Rotavirus with *Shigella* were observed most predominately in our study population. *Shigella* along with several other types of bacteria has been shown to have a recycling mechanism for their peptidoglycan, which is designed to prevent its release and interact with Nod1 [43]. In our study we found *Shigella* infection with different other diarrheal bacterial pathogens that may be the possible reason for disease severity in the patients.

At cellular and molecular level, diarrhea is simply an altered movement of ions and water. Enteric pathogens however can alter this balance towards net secretion. Together, co-infection might cause more severe diarrhea than infection with either pathogen alone [42]. Specific co-infecting pathogens might also act synergistically resulting in even greater pathogenesis and a large contribution to an overall diarrheal disease burden. In our study, we found high prevalence of co-infection similar to the other studies reported previously [41–44]. Rotavirus is mainly responsible for decreased absorption without any significant intestinal inflammation, *Shigella* species causes inflammatory and invasive diarrhea and EPEC alters the intestinal epithelial integrity [43]. We observed several mixed infections with all these three pathogens. On an average >10 diarrheal episodes/day in cases with concurrent infections might be due to an aggravated effect and a number of cellular mechanisms activated simultaneously by various pathogen factors. A true understanding of pathogenesis of diarrheal disease is incomplete without the thorough understanding of biological connections of these pathogens and synergistic interaction between co-infecting pathogens. We may be able to improve our understanding of the pathogenic potential of enteric infection consistently by distinguishing between single and mixed infection.

It is also important to include healthy controls in order to compare the distribution of exposure in healthy controls compared to cases as recommended elsewhere [35]. The overall result in our study showed that 20.83% cases in non-diarrheal children also had at least one infectious agent as detected in the stool specimens. Reports originated from other studies also showed the detection of some of those agents in stool samples from human subjects with no symptoms of diarrhea [25, 26]. In the non-diarrheal group, only 3 (6.25%) subjects were found positive for multiple infections. However, it is not clear why those apparently normal individuals had the infectious agents but no symptoms of diarrhea was noticed. However, the frequency of detection of any diarrheal infectious agent or multiple agents in these apparently healthy subjects were much less, compared to those in case of the symptomatic subjects as supported on statistical analysis.

The overall finding on a broad spectrum of etiological agents of diarrhea and different combinations of concurrent infections in the pediatric patients will probably aid in planning future studies on various aspects of diarrheal diseases in this population. It is hypothesized that in cases with concurrent infections, different combinations of etiological agents might be complementing each other’s strategies of pathogenesis resulting in an increased disease severity, and other complications, which is a matter of concern.

## Conclusion

This hospital-based study highlighted the overall burden of major bacterial, viral, and protozoan parasites in childhood diarrhea in the study region where multiple infections in nearly one-third of the cases pose a significant question to understand the synergistic role contributed by each associated pathogen in the overall pathogenesis of diarrheal diseases. Nevertheless, the results suggested that DEC strains such as EPEC and STEC, which are normally not screened routinely, should also be suspected in childhood diarrhea. Suspecting possible multiple infectious etiologies and diagnosis of the right causative agent(s) can help in a better pharmacological management of acute childhood diarrhea. Also it is recommended to address the issues of combined pathologies of co-existing diarrheagenic agents in experimental infection studies.

## Additional files

**Additional file 1.** Additional figures.

**Additional file 2.** Additional tables.

## Abbreviations

CI: confidence interval
DEC: diarrheagenic *Escherichia coli*
EPEC: enteropathogenic *Escherichia coli*
EHEC: enterohemorrhagic *Escherichia coli*
STEC: shiga toxin-producing *Escherichia coli*
EAEC: enteroaggregative *Escherichia coli*
PCR: polymerase chain reaction
WHO: World Health Organization
