# Supplementary material for: Multiple etiologies of infectious diarrhea and concurrent infections in a pediatric outpatient-based screening study in Odisha, India
Source: Gut Pathog. 2017 Apr 11;9:16. doi: 10.1186/s13099-017-0166-0 (PMC5387278; doi:10.1186/s13099-017-0166-0)
Supplement: Supplementary file 1 — Additional file 1. Additional figures. [file 13099_2017_166_MOESM1_ESM.docx]

**ADDITIONAL FIGURES**

**
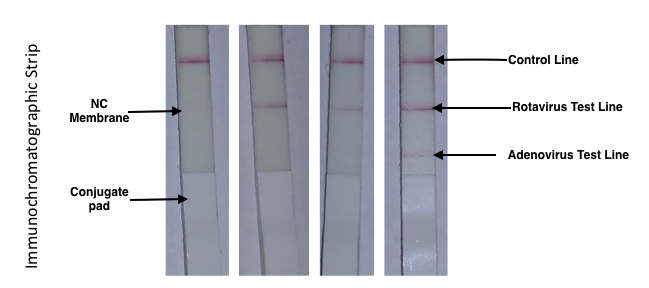
**

**A B C D**

**Additional Figure-1:** Representative picture of Immunochromatographic test.

**Negative Test Result**-Upper reddish-purple line appears at the Control line in upper most reason no other band is present (A).

**Positive Test Result-**

In addition to reddish-purple band at the control line a visible reddish-purple line after top most red line indicated *Rotavirus* positive test (B & C).

Appearance of 3 lines on strip indicates both *Rotavirus* and *Adenovirus* positive test (D).

***Note- Intensity of the test line may vary according to the quantity of antigen found in the sample.***


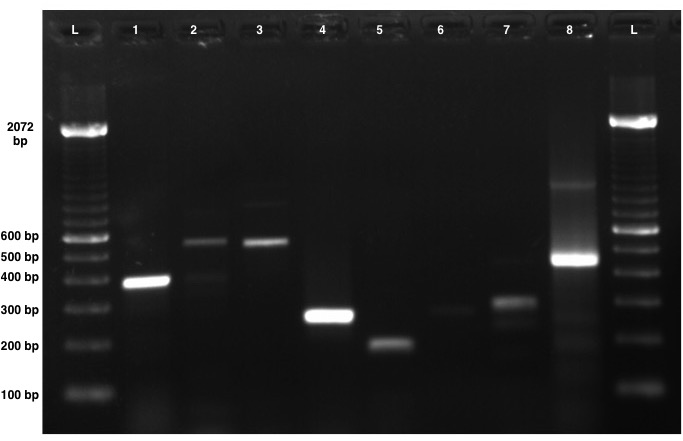


**Additional Figure-2:** Gel image of representative samples tested positive by PCR employing species-specific primer sets

Primer information is provided in Table-1 in the main manuscript.

Lane 1 to 9 sample sequences are given below-

L- 100 bp ladder

1. EPCE positive sample
2. *Shigella* positive sample
3. EHEC positive sample
4. *E. coli* stx *2* positive sample
5. *E. coli stx 1* positive sample
6. *E. coli* O157 positive sample
7. *Cryptosporidium* positive sample
8. *Giardia* positive sample


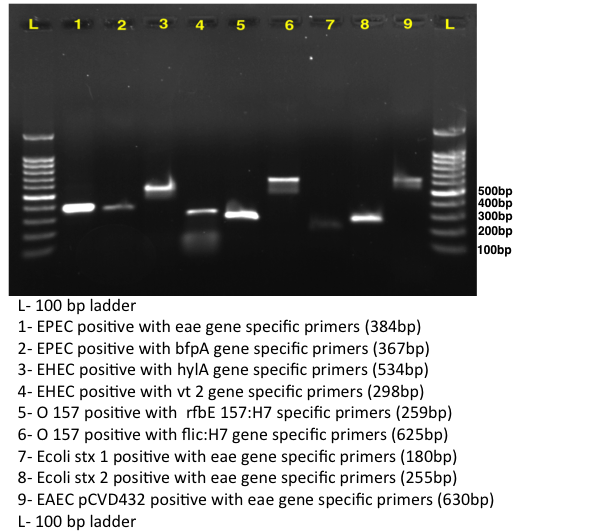


**Additional Figure-3:** Gel image of representative samples tested positive by PCR employing species-specific primer sets

Primer information is provided in Table-1 in the main manuscript.
