# Supplementary material for: Multiple etiologies of infectious diarrhea and concurrent infections in a pediatric outpatient-based screening study in Odisha, India
Source: Gut Pathog. 2017 Apr 11;9:16. doi: 10.1186/s13099-017-0166-0 (PMC5387278; doi:10.1186/s13099-017-0166-0)
Supplement: Supplementary file 2 — Additional file 2. Additional tables. [file 13099_2017_166_MOESM2_ESM.docx]

**Additional Tables**

**Additional Table-1:** Location wise distribution of incidences of infectious diarrhea due to DEC

***** Statistically significant

DEC- diarrheagenic *Escherichia coli*

Statistical analysis for significance calculation showing p-values based on one-tailed t-test (Mantel-Haenszel chi-square statistics) using free statistical software Epi-Info (http://www.openepi.com/TwobyTwo/TwobyTwo.htm).

|  | Total no. with an infectious diagnosis | Total DEC | No (%) of cases tested positive for different etiologies | | | | | |
| --- | --- | --- | --- | --- | --- | --- | --- | --- |
|  |  |  | STEC | EPEC | EHEC | O 157 | EAEC | |
| Rural | 24 | 16  (66.66) | 8  (33.33) | 12  (50) | 1  (4.16) | 3  (12.5) | 4  (16.66) | |
|  |  |  |  |  |  |  |  |  |
| Urban | 50 | 24  (48) | 6  (12) | 16  (32) | 0  (0) | 3  (6) | 5  (10) | |
| Statistics | P value | 0.0671 | * 0.0146 | 0.0688 | 0.0745 | 0.1704 | 0.2073 | |
|  | 95% CI  (lower, upper) | 42.78, 64.93 | 11.49, 29.41 | 27.63, 49.24 | 0.0, 7.973 | 3.46, 16.89 | 6.315, 21.74 | |
|  | Odds ratio | 2.167 | 3.667 | 2.125 | NA | 2.238 | 1.8 | |
